# Supplementary material for: The Presence of T Allele (rs35705950) of the MUC5B Gene Predicts Lower Baseline Forced Vital Capacity and Its Subsequent Decline in Patients with Hypersensitivity Pneumonitis
Source: Int J Mol Sci. 2023 Jun 28;24(13):10748. doi: 10.3390/ijms241310748 (PMC10341926; doi:10.3390/ijms241310748)
Supplement: Supplementary file 1 [file ijms-24-10748-s001.zip › ijms-2455420-supplementary.pdf]

Table S1. Incident and prevalent cases baseline characteristics

**Variable**	***Incident cases N=28***	***Prevalent cases N=58***	**p value**
FVC (L), median (range)	3.035 (1.985-4.310)	2.57 (2.120-3.515)	0.4648
FVC (% pred.), mean (±SD)	82.79 (±16.01)	77.48 (±20.31)	0.2425
TLco (% pred.), median (range)	59 (44-63)	54.50 (47.25-65.75)	0.9534
6MWD (m), mean (±SD)	492.6 (±110.8)	509.4 (±112.7)	0.5382
DSP, mean (±SD)	438.6 (±114.4)	469.2 (±93.59)	0.2137
GT/TT, N° (%)	7 (25)	19 (32.76)	0.6173
